# Refining the Implementation of a Hub-and-Spoke Model for TelePain Through Qualitative Inquiry

**DOI:** 10.1007/s41347-022-00288-w

**Published:** 2022-12-06

**Authors:** Soumya Subramaniam, Jessica Chen, Tai-Lyn Wilkerson, Lauren Stevenson, Carrie Kincaid, Christine Firestone, Sherry L. Ball

**Affiliations:** 1 VA Northeast Ohio Healthcare System, 10701 East Blvd, Cleveland, OH 44106 USA; 2 Puget Sound VA Healthcare System, 1660 S, Columbian Way, Seattle, WA 98108 USA

**Keywords:** Pain management, Telehealth, Veterans healthcare, Care delivery model, Chronic pain, Interdisciplinary care

## Abstract

The hub-and-spoke telehealth model leverages centrally located providers who utilize telehealth technology to bring specialized care to medically underserved areas. This model has the potential to promote equitable access to healthcare. However, few studies address how to facilitate the adoption and implementation of hub-and-spoke telehealth. We examined spoke site providers’ experiences with TelePain, a national hub-and-spoke model of interdisciplinary chronic pain care, with a focus on improving future implementation. We conducted semi-structured individual interviews (20–45 min) with 27 VA spoke site providers via teleconferencing between August 2020 and February 2021. Interview transcripts were coded in Atlas.ti 8.0 using deductive (identified a priori and used to build the interview guide) and inductive (emerging) codes. Our analysis identified the following themes stressed by the spoke sites: (1) spoke sites needed to envision how TelePain services would work at their site before deciding to adopt; (2) TelePain implementation needed to fit into local existing care processes; (3) hub sites needed to understand spoke sites’ context (e.g., via needs assessment) to tailor the services accordingly, and (4) hub-and-spoke sites needed to establish bidirectional communication. Our findings provide a practical guide to improve future rollout of hub-and-spoke telehealth models. Recommendations focus on the role of the hub site in promoting program adoption by (1) developing a clear and detailed marketing plan and (2) considering how the program can be adapted to fit the local spoke site context. To improve implementation, hub-and-spoke sites must establish ongoing and consistent bidirectional communication; this is particularly critical in the everchanging post-peak pandemic healthcare system. An important next step is the development of recommendations and guidelines for implementing hub-and-spoke telehealth, as well as examining pain outcomes for patients touched by this program.

## Introduction

Hub-and-spoke telehealth models address shortages of specialists in medically underserved areas, particularly rural areas (Demaerschalk et al., 2013; Stingley & Schultz, 2014). In a hub-and-spoke telehealth model, specialists located in larger healthcare facilities (e.g., academic medical centers) use video telehealth, telephone, and asynchronous telehealth (e.g., remote monitoring, secure messaging, and other methods where the provider and patient do not engage in the clinical encounter at the same time) to deliver services to patients participating from spoke clinics in their community or from home, thereby increasing access to specialty care. Application of this model, used in acute stroke care, emergency medicine, neurology, oncology, mental health, plastic surgery, social work, rheumatology, and pain ((Demaerschalk et al., 2013; Glynn et al., 2021; Miele et al., 2020; Scalise et al., 2015; Schreck et al., 2020; Stingley & Schultz, 2014), has resulted in improved efficiency, access, and cost-effectiveness (Chen et al., 2022; Demaerschalk et al., 2013; Elrod & Fortenberry, 2017a). Emerging data regarding barriers and facilitators to implementation have focused on opioid use disorder (Caton et al., 2019; Snell-Rood et al., 2021), while the implementation of other specialty care hub-and-spoke models is understudied. Therefore, healthcare systems need more guidance for replicating or expanding this model of telehealth.

Chronic pain, a leading cause of disability worldwide (Vos et al., 2017), disproportionately impacts veterans and military personnel (Nahin, 2017). Chronic pain increases the risk of opioid overdose and suicide (Bohnert, 2011; Ilgen et al., 2013; Zedler et al., 2014). Rural areas have limited access to evidence-based, biopsychosocial chronic pain treatment, specifically interdisciplinary pain management teams (e.g., a physician, a physical therapist, and a psychologist who work together to provide medication, exercise treatment, and cognitive behavioral therapy (Institute of Medicine, 2011; U.S. Department of Health and Human Services, 2019)). Veterans living in rural areas are less likely to access specialty pain services (Arout et al., 2017), are prescribed 30% more opioids, and have higher rates of opioid misuse and mortality (Lund et al., 2019) than their urban counterparts. The use of hub-and-spoke pain care can improve access and therefore outcomes for patients living with chronic pain.

To address gaps in pain care in rural areas, the Veterans Health Administration (VA) is implementing a hub-and-spoke telehealth model (Brecht et al., 2020), TelePain, to bring interdisciplinary pain care to sites that lack their own specialty pain management teams. Previously published literature has described the process for setting up a TelePain program (Glynn et al., 2021); observed increased access to pain care and increased use of telehealth for pain among rural patients following the implementation of TelePain (Chen et al., 2022); and documented patient satisfaction with the TelePain model of care (Silvestrini et al., 2021). In the VA TelePain model, a hub located at a major VA medical center hosts an interdisciplinary pain team that delivers multimodal, biopsychosocial pain service to spoke sites, which are rural community-based outpatient clinics and small VA facilities in the region. TelePain hubs include a program manager, physicians trained in pain medicine, clinical pharmacists, nurses, physical therapists, psychologists, social workers, telehealth clinical technicians, and medical support assistants. Services offered include evaluation of pain conditions, chronic pain education, medication management, and psychosocial and mind–body treatments for chronic pain. TelePain adds nonpharmacological treatment options, such as evidence-based psychotherapies (e.g., cognitive behavioral therapy for chronic pain). TelePain also differs from a multidisciplinary treatment model where patients see a primary care physician, a mental health clinician, and a physical therapist separately. Instead, the interdisciplinary team model supports the integration of medication management with physical and behavioral therapy, all embedded within critical pain education that de-medicalizes the chronic pain experience and focuses on improved functioning (Boon et al. 2004; Gatchel et al., 2014). Ample evidence supports that successful chronic pain intervention relies on changing patient perceptions and beliefs about pain (Jensen et al., 2007; Lee et al. 2015) Add citations). Without TelePain, pain care at spoke sites is typically handled by a combination of primary care providers, who rely on e-consults to specialists; mental or behavioral health providers embedded in primary care, specialty care, or non-VA community providers, who may address depression, anxiety, or co-occurring mental health disorders but generally do not offer evidence-based treatment for pain; and community pain providers, who generally focus on biomedical interventions such as spinal injections.

While the implementation of TelePain is the first step towards addressing access gaps, understanding best practices will be important to sustain and spread the hub-and-spoke telehealth model of specialty care. Although prior research has established the feasibility of hub-and-spoke pain care via telehealth and preliminary evidence for improved access and patient satisfaction, little is known about how to effectively implement or scale up this model of telehealth. The present study sought to identify the barriers and facilitators to implementing a hub-and-spoke model of specialty pain care, with a focus on the perspectives of those adopting this telehealth model at the spoke sites.

## Methods

We evaluated implementation of TelePain with a focus on the perspectives of spoke site providers. We interviewed clinicians at two VA TelePain sites at different stages of implementation: a pre-implementation phase (with plans to launch in the upcoming year) and a post-implementation phase (10 months after launching TelePain). At the pre-implementation site, which serves a three-state region in the southeastern USA, the goal was to assess spoke site providers’ receptivity to adopting this new model of care. At the post-implementation site, which serves a four-state region in the northwestern USA, the goal was to assess spoke site providers’ perceptions of TelePain after they had referred patients to the hub.

Typically, implementation activities are spearheaded by the TelePain clinical team and can include activities such as presenting at clinic or staff meetings to introduce TelePain to spoke site providers in primary care and behavioral/mental health; setting up monthly meetings between the TelePain team and clinic leaders at spoke sites to discuss referral flows and marketing/advertising TelePain’s services; and sharing patient education materials on TelePain services and the benefits of engagement with the program.

### Participants

The implementation was the first phase of a larger rollout of TelePain across the national VA setting. Implementation facilitators working with TelePain sites shared a list of all 57 VA providers participating or who had agreed to participate in TelePain at these two hub sites and 11 spoke sites to the qualitative team. All 57 providers were invited by email to participate in a 30-min interview about their experiences with TelePain. Union approval was obtained before pursing these interviews. The work was determined to be quality improvement by the VA Northeast Ohio Healthcare System Research and Development committee and no human subjects review was needed.

### Data Collection

Individual interviews were conducted from September 2020 to January 2021 using a semi-structured interview guide (Supplement 1) designed to elicit barriers and facilitators to TelePain adoption as well as suggested improvements to the TelePain model. We drew upon the Consolidated Framework for Implementation Research (Damschroder et al., 2009) to develop 12 interview guide questions and included open-ended, grounded probes to elicit new or unexpected information in a uniform manner while allowing for exploration of unanticipated themes generated by participants. While the interview guide was designed to elicit data from the perspective of the spoke site providers, the data collected were expected to inform ongoing and future program design and rollout by the implementation team and hub providers. Twenty-seven interviews were conducted by three experienced qualitative interviewers between August 2020 and February 2021. Interviewers had no experience working with any of the respondents and were external to the implementation team. Interviews were scheduled by a separate research coordinator based on availability of respondents and interviewers’ time schedule. After obtaining verbal consent for audio-recording from the respondent, each interview was audio-recorded. Analysis team members debriefed after each interview for quality assurance to reflect on the interview content and whether any adaptations needed to be made to the interview guide to capture relevant information. Interviews were 20–45 min each.

### Data Analysis

Audio-recordings were transcribed verbatim and transcripts were verified for accuracy. No participant names or identifiers were included. Atlas.ti software (version 8, Scientific Software Development GmbH, Berlin, Germany) was used for data management, coding, and analysis. Content analysis (Elo & Kyngäs, 2008) was used with codes corresponding to initial CFIR constructs (used to generate interview guide questions). Two researchers independently coded 27 transcripts using the initial codebook made during consensus meeting between analysts and noted any emerging codes. After comparing and discussing analyses, the initial codebook was revised to clarify existing codes and to operationalize new codes. Initial interviews were re-analyzed to include new codes. An additional qualitative analyst reviewed all transcripts to resolve discrepancies in coding and differences in interpretation. To develop themes, we used a grounded approach where the theory used to frame the data was derived from the participants’ responses (Corbin & Strauss, 1990). Supporting quotes from respondents are included in results to support and illustrate emerging themes. The authors include the use of qualifiers throughout the findings and discussion section of this paper. These qualifiers are not the result of magnitude coding but are included to enhance accuracy in data reporting (Saldaña, 2014).

## Results

### Participants

Table 1 lists the roles for all 57 participants who were contacted and all 27 participants who agreed to participate in this quality improvement project and for whom telephone interviews were scheduled and completed.

**Table 1:** Provider recruitment

**Provider type**	**Total participants (n** = 27)		**Contacted, did not enroll (n** = 30)
	**Pre-implementation (*n* = 17)**	**Post-implementation (*n* = 10)**	
*Physician*	4	2	10
*Psychologist*	4	3	3
*Nurse practitioner*	2	1	1
*Pharmacist*	2	3	3
*Whole health coach*	2		6
*Nurse*	2		1
*Telehealth coordinator*	1		
*Addiction specialist*		1	
*Physician assistant*			1
*Director/assoc. chief of staff*			2
*Unknown*			3

### Summary

Spoke site providers emphasized the need to establish strong communication mechanisms between hub-and-spoke site providers prior to implementation and to strengthen those connections between the sites throughout implementation. Respondents shared that it was critical that hub sites disseminate clear and consistent information about TelePain. At a minimum, information conveyed should describe the types of services delivered and steps for referral. Communication about TelePain should enable the potential spoke site participant to envision how TelePain services would be integrated into existing clinical care offerings. The decision to adopt TelePain (i.e., refer patients) relied on whether TelePain fit the spoke site’s needs, existing resources, and needs and preferences of their local patient population. Once a decision to adopt was made, dissemination and implementation plans needed to be adapted to fit the spoke site. In addition to conveying clear and detailed information from the hub to the spoke during adoption, establishing clear mechanisms for ongoing, two-way communication between hub-and-spoke sites throughout implementation was critical to ongoing success.

#### Program Dissemination by Hub Site Informed Spoke Site’s Decision to Adopt

Respondents shared their views about the importance of the dissemination of program materials to assist sites in making an informed decision regarding whether to adopt TelePain. Spoke site provider respondents had limited understanding of TelePain prior to implementation and expressed a need for more information and training on services offered by the hub providers. Needed information included the following: how the program would work at their site, what types of patients to refer, and how to incorporate the program into existing pain care services, including how hub-and-spoke site providers would “work together” (Table 2: quotation (Q1)). Suggestions to improve program dissemination included offering training for providers and veterans that explains how the program would complement existing resources or fill gaps.

**Table 2:** Quotations (supporting evidence) for findings

**Finding**	**Representative quotation (Q)**
Program dissemination: critical to inform decision to adopt	Q1. *it would really be nice if they would offer a training or something … I’m not real familiar with TelePain…what they do and what they offer and how they would best fit in with our practices and how we could work together* (PRE-IMPLEMENTATION_14)
	Q2. *If we try to do something … that requires primary care to do a lot of clicking or entering of consults, it’s not going to do anything but cause more burnout*” (PRE-IMPLEMENTATION_18)
	Q3. *With a lot of veterans … they feel that they don’t get enough information upfront, … they’re not told a lot of detail about the treatment that they’re undergoing in terms of managing pain and so it’s not until later on or maybe from someone else like a psychologist versus the prescriber that they’re receiving more detailed information and so that would be very helpful.* (PRE-IMPLEMENTATION_012)
	Q4. *Yeah, just thinking about like a handout or a video or some kind of format like that would be would be useful for patients.I would say if there’s an electronic type format because of all our waiting rooms you know have uh, have the TVs in them..* (PRE-IMPLEMENTATION_15)
	Q5. *I like YouTube videos for patient education …[for] the younger guys its very helpful. You know they like everything electronic, they don’t like paper. And so, you have to customize it, you have to be able to do all of the above.* (PRE-IMPLEMENTATION_17)
	Q6. *maybe mail the patient something eductational once they get referred to TelePain.* (PRE-IMPLEMENTATION_18)
Decision to adopt informed by patient needs and preferences	Q7. *… if we could offer some sort of alternative here.. through the TelePain services, it would be beneficial cause then they don’t have to travel all the way to [X], which is again you know 80 miles away and it kind of negates the treatment they get when they’re there by the… time they’re back home.* (PRE-IMPLEMENTATION_24)
	Q8. *veterans…in our Whole Health type clinic that actually want to get off the opioids … their complaints are more the fact that we don’t have as many complementary services as they would like to see.* (PRE-IMPLEMENTATION_15)
	Q9. *even though we try to address pain with the standards of care, you really have to look at that person individually and come up with a plan of care that fits them.* (PRE-IMPLEMENTATION_11)
	Q6. *the veteran herself had done a lot of that [yoga and tai-chi] in the past and that got her really excited about how people who are trained in pain management are doing things that she cares about and loves so it just gave her some hope that there might be something out there.* (POST-IMPLEMENTATION_10)
Decision to adopt informed by challenges to patient participation	Q10. *I have quite a few that just don’t feel comfortable with anything that is not face to face. There are some that have hearing issues …. It tends to be the older, especially more remote rural veterans that are less comfortable with technology in general but some are middle age mental health veterans who really just are creeped out I guess by the video chatting experience.* (POST-IMPLEMENTATION_03)
	Q11. *this guy [*veteran*] said where he’s from, he’s in the woods, … he has no access to, no internet, no technology…basically the infrastructure, right?* (PRE-IMPLEMENTATION_17)
Decision to adopt informed by needs of individual spoke sites	Q12. *I just saw the flyer … so it sounds like a good way for somebody to … have the time to go through what might work for a veteran and give some recommendations…But I’ll tell you that our primary care providers are overworked and overburdened and burned out. So if we try to do something … that requires primary care to do a lot of clicking or entering of consults, it’s not going to do anything but cause more burnout.* (PRE-IMPLEMENTATION_18)
	Q13. *for our veterans that do have pain, integrate at least one visit where the TelePain provider [is] on the screen as well as their primary care provider and the veteran so that everybody can kind of be on the same page as to what is our goal, what are we trying to do*. (PRE-IMPLEMENTATION_15)
	Q14. *We don’t have, currently have an interdisciplinary team, which I think would be beneficial to the patients. We kind of have each provider addressing their own piece of it but that always doesn’t interpret into a cohesive plan.* (PRE-IMPLEMENTATION_21)
Importance of communication with champion and leadership	Q15. *It's a must to have somebody locally build that relationship … as a long-term strategy. I think anytime that you can have a meeting between a champion and the actual staff to troubleshoot issues, I think it’s useful. I have found over the years that when we don’t communicate things…items just don’t get done or there’s information that’s miscommunicated or misconstrued and it ends up making the team not as productive.* (PRE-IMPLEMENTATION_19)
	Q16. *There definitely needs to be a way for us [spoke site] to communicate to you guys [hub site] if a veteran has a complaint or an issue…or if we have a follow up meeting every quarter or…like somebody we can have as a contact person like a clinical champion if I have a problem, that’s easier than having a meeting. I mean, we can have a meeting but most of the clinicians aren’t going to have blocked out time to have a meeting.* (PRE-IMPLEMENTATION_18)
Clarifying roles	Q17. *my patient at least was misconstruing what your [hub site] role was, she had thought that you were going to be talking with her primary physician about other pain options and then it didn’t seem like that happened, so I think all of it just ended up being some miscommunication on my part as well. So, I think I could have been more direct in asking what this program is, what are you doing, how are you helping, that sort of thing.* (POST-IMPLEMENTATION_10)
	Q18. … *sometimes it would help to have that second opinion or maybe there’s something that we haven’t thought about or a treatment that we're not aware about because we don’t have a specialist here that would perform that procedure*. (PRE-IMPLEMENTATION_21)
	Q19. *I would want to call on uh TelePain if I had reached capacity or if there was some sort of intervention that I thought the, the Vet would really benefit from that I didn’t have the expertise to offer myself.* (PRE-IMPLEMENTATION_26)
	Q20. *Some more like direct education from the providers in the TelePain clinic on what they have available, what they do and the rationale for that and how it’s to refer patients and maybe just more regular interfacing with like our local leadership or stakeholders like PCMHI or the pain team here to coordinate any particularly like complicated veteran cases and things like that. So increasing that collaboration instead of having more primarily like a treatment referral service.* (POST-IMPLEMENTATION_08)
Consult Process	Q21. “*they let the veteran know at the end of the visit the recommendations, … the recommendations would go in, even at real time. [At] The time of the appointment we had enough information to kind of change the course of therapy and it was a good positive change*.” (POST-IMPLEMENTATION_09)
Clarifying Communication	Q22. *I’d probably say more routine or formal feedback on the patients and their progress and corroboration in that regard. Cause I’ve had some patients that they went like initial intake appointment, and they weren’t sure if they wanted to follow up and kind of left it there….* (POST-IMPLEMENTATION_08)
	Q23. *The medical provider has different information than I [mental health specialist] have and so more of the team approach at least especially with pain because it’s you know, it has so many different dynamics to it, kind of that holistic approach where everybody’s looped in.* (POST-IMPLEMENTATION_05)
Implementation Success: Improving Perceived Access to Pain Care	Q24. *“[TelePain]’s definitely a benefit and I would personally, I think, my [clinic] and colleagues would benefit knowing more about the program, and maybe just [be] reminded that [it] exists and what you all are able to offer, having some kind of handout to be able to give to the patient.* (POST-IMPLEMENTATION_07*)*
	Q25: “*It’s improved access because even here locally, non-VA wise, we really don’t have any pain specialist…like we only have very few psychologists in our community and none of them are “pain.” You know that’s not their forte…so it’s definitely, I think improved access to pain care.* (POST-IMPLEMENTATION_03)
	Q26: *Oh definitely it [TelePain] feels like it opened it up. I think that overall our pain services feel like they’re a lot more robust than they were when I first started here and I suspect the other providers are working more collaboratively with the TelePain services cause it’s opened a lot of different options and services here at our facility, too…I don’t know if it is a result of partnering with TelePain, but it’s definitely been a really helpful resource for veterans who are not thrilled about trying these other nonmedication option. I think they always look for someone to vent about it too which I know that’s not what therapy is, but the thought of having that external support to get through it, I think is really beneficial, so it’s been good.* (POST-IMPLEMENTATION_04)

A barrier to implementation was that respondents expressed concerns that referring to TelePain would impact spoke site primary care provider (PCP) workload, stating “If we try to do something … that requires primary care to do a lot of clicking or entering of consults, it’s not going to do anything but cause more burnout” (Q2). Respondents sought more clarity around the exact toll on referring providers if they were to engage an external hub-and-spoke program. Although the intention of TelePain is to reduce PCP workload by having tele-specialists take over pain management for more complex patients, it was clear that hub sites needed to convey this message very clearly to gain spoke site buy-in.

#### Spoke Site’s Decision to Adopt Informed by Patient Needs and Preferences

Data from respondents suggest that a deliberate and clear consideration of patients’ needs and existing resources facilitates adoption. Spoke site providers interviewed prior to implementation highlighted the benefits of a telehealth program: “it would be beneficial cause then they don’t have to travel all the way to [X], which is again you know 80 miles away” (Q3). They also mentioned fit between TelePain services and patients’ requests, such as wanting more nonpharmaceutical approaches instead of narcotics (Q4). Respondents shared that TelePain could help create individualized care plans that fit veterans’ pain care needs and would help patients make informed healthcare decisions (Q5). One respondent interviewed post-implementation shared the enthusiasm for TelePain services from one veteran (Q6).

#### Spoke Site’s Decision to Adopt was Informed by Challenges to Patient Participation

There were challenges to getting veterans to participate in TelePain. One provider perceived that some veterans do not feel comfortable with virtual platforms and would prefer face-to-face care (Q7). One respondent noted that some veterans may not have the needed internet access because of their rural location (Q8). Providers discussed how veterans’ buy-in was also crucial to implementation. Veterans needed detailed information about evidence-based pain care up front (Q9), and respondents suggested creating handouts or videos to explain TelePain services (Q10–12) and chronic pain (Q9–12) to patients.

#### Spoke Site’s Decision to Adopt Informed by Local Needs

Respondents suggested how TelePain could be integrated into existing primary care clinics to provide a platform for interdisciplinary communication that facilitates coordinated care, such as including “at least one visit” between the TelePain provider, the primary care provider, and the veteran “so that everybody can…be on the same page as to what is our goal, what are we trying to do” (Q13). For clinics or facilities that lacked an interdisciplinary pain team, respondents highlighted how the TelePain model could improve the cohesion of pain care (Q14).

#### Hub-and-Spoke Sites Communicate and Share Information with Clinical Champions and Leadership

During pre- and post-implementation, respondents speculated on the importance of identifying clinical champions at each spoke site to facilitate communication and placed emphasis on obtaining leadership buy-in prior to implementation to foster program adoption, stating “It’s a must to have somebody locally build that relationship” (Q15) and “somebody we can have as a contact person like a clinical champion if I have a problem” (Q16). During post-implementation, respondents highlighted the need to clarify roles and improve communication between the referring provider, the veteran, and TelePain hub team members (Q17).

Respondents were split in whether they wanted to use TelePain as a consult to support the primary care team, who would remain the veteran’s primary point of contact, or to “handoff” complex cases to specialty providers so that spoke site providers could be less overwhelmed (Q18–Q20).

Two respondents stated at post-implementation that the real-time group consult worked well, and they felt that the recommendations of the TelePain team reinforced what the site PCPs had told their patients. One respondent said that it was “good to have backup” in the veteran’s care. Another provider commented on the efficiency of the TelePain team at post-implementation, “they let the veteran know at the end of the visit the recommendations, … the recommendations would go in, even at real time. [At] The time of the appointment we had enough information to kind of change the course of therapy and it was a good positive change.” (Q21).

Spoke sites expressed a need for updates and reminders about services offered through TelePain and a strong desire to receive follow-up information from the TelePain hub providers about whether and how veterans followed up with any recommendations and/or care. Respondents suggested that routine feedback from TelePain providers on patient participation in services and progress is “necessary” and would “close the loop” (Q22). Respondents suggested that it would be useful to get more information regarding the role of the TelePain team to improve communication and have a meeting with the team prior to the consultation (Q23).

### Improving Access to Pain Care

Participants felt that TelePain was beneficial for them and their patients because it improved access to care for veterans, especially those in rural areas (Q24). One provider stated “It’s improved access because even here locally, non-VA wise, we really don’t have any pain specialist” (Q25). Another remarked “Overall our pain services feel like they’re a lot more robust than they were when I first started here and I suspect the other providers are working more collaboratively with the TelePain services cause it’s opened a lot of different options and services here at our facility” (Q26).

## Discussion

This qualitative project explored spoke site providers’ experiences with a VA hub-and-spoke model of specialty pain care, TelePain. This exploration was carried out in partnership with hub site leaders and was designed for hub sites to learn directly from spoke site providers about directions for program improvement. Most spoke provider respondents felt that TelePain was beneficial for them and their patients, speculating that it improved access to, lowered costs associated with, and improved veteran experiences with pain care, especially for those residing in rural areas. These findings align with the literature regarding the experience of telemedicine and it’s adoption within the VA and for primary care and adds to them with the addition of the hub-and-spoke model (Connolly et al., 2022; Der-Matirosian et al. 2021; Myers et al., 2020). The hub-and-spoke model provides sustainable specialty care to remote regions (Elrod & Fortneberry, 2017b; Rawson et al., 2019; Switzer et al., 2013). These perceptions are in line with those previously reported in evaluations of VA’s TelePain (Chen et al., 2022) and other research examining hub-and-spoke models (Devarakonda, 2016; Silvestrini, Indresano, Chen 2022,Lin et al., 2021). Our findings support existing studies and offer some suggestions to improve implementation of hub-and-spoke programs.

Our findings highlight the need to carefully consider three key components when implementing a hub-and-spoke model: program dissemination by the *hub*, the unique context, and characteristics of individual *spoke* sites and *connectors* or how the hub-and-spoke sites will communicate and share information. Program success may be improved when *hub* sites distribute clear and comprehensive information about services offered. The hub site should craft clear marketing materials, such as patient and provider flyers, and develop a dissemination plan that reflects a strong understanding of the spoke sites’ needs and resources including each spoke sites’ patient population. Based on the materials disseminated by the hub site, the spoke site should have a good picture of how the program could work in their clinic. The hub site carries the initial onus of understanding the *needs and resources of the spoke site* and to adapt the program to fill gaps in existing services and integrate into existing programs (Green et al., 2021; Lesher et al., 2020; Snell-Rood et al., 2021). Patients have unique beliefs and behaviors that will influence decision making around pain care treatment (Bair et al., 2009; Matthias et al., 2017; Purcell et al., 2019). Spoke sites depend on information disseminated by the hub and their local site’s need to make the final decision to adopt the program.

Connections between hub-and-spoke sites are strengthened when there is a continual process of knowledge sharing and information exchange. In this case, hub leaders are responsible for program dissemination to facilitate spoke site providers’ awareness. Structures for ongoing, back-and-forth communication between the hub and spoke facilitate behavior changes needed for program adoption and implementation.

**Fig. 1:**
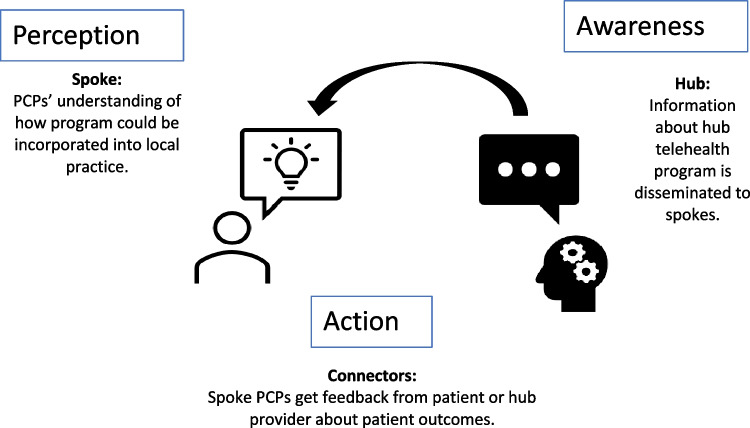
The sensemaking framework illustrates how spoke site providers can adopt and implement a new program into their work through a process of becoming aware of the program, perceiving how it will fit into their work and continue to participate or take action based on program feedback

Sensemaking theory may help us understand the data, as it suggests that successful behavior change needed for program adoption and implementation involves multiple steps including awareness, perception, and action (Fig. 1). Awareness starts with the hub site’s responsibility for understanding the needs and resources of local sites and communicating in a way that promotes the spoke site’s perception of the program. Once hub providers have gained a full *awareness* and understanding of the spoke site’s needs and resources, information about TelePain can be disseminated to the spoke site. Spoke sites can form their own *perception* of the program by envisioning local program implementation and determining whether there is a need for such services at their local site. Spoke site providers will imagine how the program could be incorporated into their existing daily workflow. However, providers may hesitate to participate in any program that feels like an additional daily task. Thus, emphasis should be placed on how the program can ease the burden of PCPs while improving patient care.

The importance of strong connectors between hub-and-spoke sites illustrates the *action* arm of the sensemaking framework. At post-implementation, spoke site providers must reflect on whether the program is yielding desirable results. The action begins as an experimental phase when feedback and strong communication or connectors between hub and spokes can be strengthen by clarifying roles, establishing clear processes such as consultation processes. Engaging in ongoing improvements in the processes by which hub-and-spoke sites communicate can facilitate program implementation.

### Limitations

This project focused on the experiences and perspectives of spoke site providers to inform how a hub site could more effectively implement a hub-and-spoke model of specialty care. No data were collected from hub providers. Other studies from the perspective of the hub provider reported increases in the number of patients that a hub site can serve and increased provider satisfaction in providing care (Guttierrez et al., 2021; Shayevitz et al., 2021).

No systematic comparisons were made between data collected during pre- and post-implementation time points. Rather, some commonalities were noted that support the conclusions. We included interviews from two VA specialty care hub-and-spoke programs. Only spoke site providers who were known to TelePain were offered a chance to interview. The interviews were conducted when many providers were distracted by the COVID-19 pandemic. It is likely that there are many other barriers to both learning about the program and participating in the program that are not included in this sample.

Although the COVID-19 pandemic forced a rapid increase in the use of telemedicine (Wosik et al., 2020), the VA had previously recognized the benefits of telemedicine (Russo et al., 2016). In this project, some PCP participants felt that many veterans were hesitant to use video-conferencing technology for medical appointments. However, prior studies suggest that many veterans found video-based healthcare to be similar to or better than in-person care (Slightam et al., 2020), and with the increase in use of telemedicine triggered by the pandemic, more veterans and other patients may be experiencing increased comfort with telemedicine (Tenforde et al., 2020).

## Conclusions

The process of sensemaking can inform the adoption and implementation of a new program. Spoke site providers first need to gain an awareness and understanding of the hub-and-spoke program that will enable them to envision how the program will fit into their existing patient care services and make a decision regarding adoption. Once a decision to adopt a program has been made, communication and feedback between hub-and-spoke site providers will facilitate program implementation. As this program expands across the country, ongoing studies will monitor effects on access to pain care and patient outcomes related to pain management.
